# Stepwise multi-gate control of the HOG MAPK pathway under hyperosmotic stress

**DOI:** 10.1016/j.isci.2026.116132

**Published:** 2026-05-28

**Authors:** Kazuo Tatebayashi, Haruo Saito

**Affiliations:** 1 Laboratory of Molecular Genetics, Frontier Research Unit, Institute of Medical Science, The University of Tokyo, Tokyo 108-8639, Japan; 2 Department of Biological Sciences, Graduate School of Science, The University of Tokyo, Tokyo 113-0033, Japan; 3 Division of Molecular Cell Signaling, Institute of Medical Science, The University of Tokyo, Tokyo 108-8639, Japan

**Keywords:** Molecular biology, Cell biology

## Abstract

In the budding yeast *Saccharomyces cerevisiae*, adaptation to hyperosmotic stress is mediated by the Hog1 mitogen-activated protein kinase (MAPK) via the high-osmolarity glycerol (HOG) pathway, which comprises a MAPK cascade and two upstream branches, SHO1 and SLN1. In the SHO1 branch, hyperosmotic stress is detected by transmembrane proteins such as Sho1, Opy2, Hkr1, and Msb2; however, the signaling steps directly controlled by the stress have remained unclear. Here, we show that hyperosmotic stress regulates three distinct steps within the SHO1 branch. It promotes the phosphorylation of the MAP2K Pbs2 by the MAP3K Ste11 through Sho1-dependent protein interactions and requires Hkr1, and it also regulates an upstream step required for Ste11 activation that depends on Hkr1 and Opy2. Together with a previously described step in Pbs2-mediated Hog1 phosphorylation, these findings show that osmotic stress regulates the pathway at three levels, defining stepwise multi-gate control of HOG pathway activation.

## Highlights

• Hyperosmotic stress controls the HOG pathway via three step-specific gates
• Multi-step gating ensures strong Hog1 activation only under hyperosmotic stress
• Pre- and post-Ste11 steps are controlled by osmosensor-mediated interactions
• Hkr1, but not Msb2, functions as a predominant TM mucin osmosensor

## Graphical abstract

**Figure:**
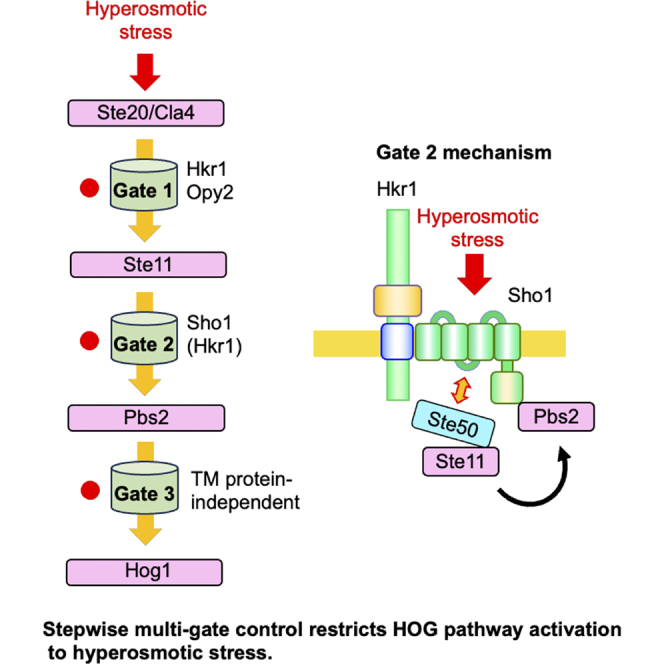

## Introduction

To cope with extracellular hyperosmotic stress, the budding yeast *Saccharomyces cerevisiae* activates the Hog1 mitogen-activated protein kinase (MAPK) through the high osmolarity glycerol (HOG) pathway. Activation of Hog1 triggers a broad spectrum of adaptive responses. These include the synthesis, uptake, and intracellular retention of the compatible osmolyte glycerol, as well as extensive changes in transcription, translation, and cell-cycle progression.^1^^,^^2^^,^^3^^,^^4^ These responses are essential for maintaining cellular homeostasis and viability under conditions of high environmental osmolarity.

**Figure 1:**
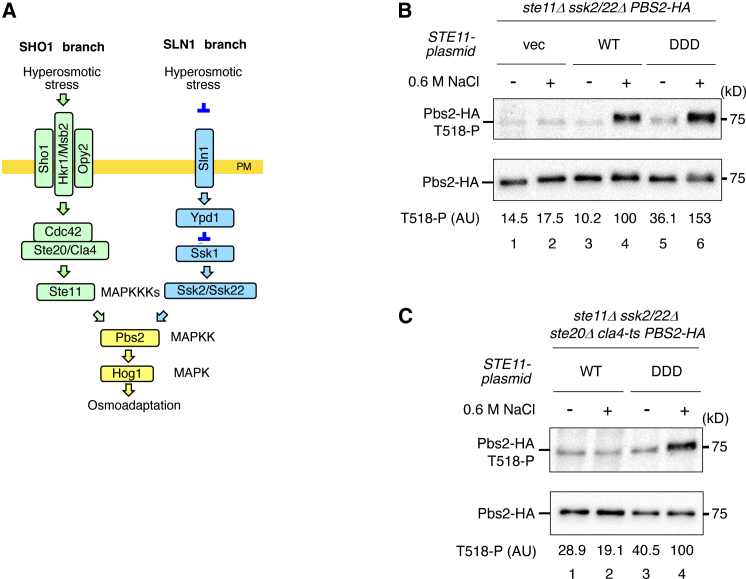
Hyperosmotic stress enhances Pbs2 phosphorylation by activated Ste11 independently of Ste20/Cla4 (A) Schematic model of the HOG pathway. SHO1-branch-specific proteins are shown in light green, SLN1-branch-specific proteins in light blue. Pbs2 and Hog1 are common to both branches. Proteins separated by a slash (/) are functionally redundant. The yellow horizontal bar represents the plasma membrane (PM). Arrows indicate activation; inverted T-shaped bars indicate inhibition. (B) Immunoblot analysis of Pbs2 phosphorylation at Thr518. KT320 (*ste11Δ ssk2/22Δ pbs2Δ*) was transformed with YCplac22I′-Pbs2-HA together with pRS416, pRS416-STE11-WT, or pRS416-STE11-DDD. Cells were treated with 0.6 M NaCl or left untreated for 5 min. Immunoprecipitation and immunoblotting were performed as described. Phospho-T518 Pbs2-HA levels were normalized to total Pbs2-HA. AU, arbitrary units. vec, vector; WT, wild type; DDD, S281D/S285D/T286D. (C) As in (B), KT396 (*ste11Δ ssk2/22Δ ste20Δ cla4-ts PBS2*) was transformed with YCplac22I′-Pbs2-HA and pRS413-STE11-WT or -DDD. Cells were grown at 25°C and shifted to 37°C for 1 h prior to NaCl treatment. Immunoprecipitation, immunoblotting, and quantification were performed as described in (B). Representative immunoblots from at least two independent experiments are shown. Densitometric values are shown for the same representative experiments.

The HOG pathway consists of a conserved MAPK cascade and two upstream, parallel osmosensing branches—designated the SHO1 and SLN1 branches—that converge on the MAPK kinase (MAP2K) Pbs2 to activate Hog1 (Figure 1A).^2^ In the SLN1 branch, whose signaling mechanism has been well characterized, the transmembrane (TM) histidine kinase Sln1 functions as an osmosensor and transduces osmotic information through a phosphorelay involving Ypd1 and Ssk1, leading to the activation of the functionally redundant MAPK kinase kinases (MAP3Ks) Ssk2 and Ssk22.^5^^,^^6^ Activated Ssk2/22 phosphorylates Pbs2 on both Ser514 and Thr518, thereby enabling full activation of Hog1.

In contrast, the SHO1 branch is less well understood and is thought to rely on multicomponent osmosensing complexes composed of TM proteins Sho1, Opy2, Hkr1, and Msb2.^7^^,^^8^^,^^9^^,^^10^^,^^11^^,^^12^ In this branch, hyperosmotic stress is transmitted to Pbs2 through the activation of the MAP3K Ste11 by the p21-activated kinases (PAKs) Ste20 or Cla4.^13^^,^^14^^,^^15^^,^^16^^,^^17^ Activated Ste11 phosphorylates Pbs2 only on Thr518, generating a monophosphorylated form of Pbs2 that is competent to activate Hog1 under appropriate conditions.^18^

Although Hog1 activation is classically associated with hyperosmotic stress, Hog1 can also be activated by several non-osmotic stresses through mechanisms that differ from osmotic signal transmission. For example, oxidative stress induced by hydrogen peroxide (H_2_O_2_) results in modest Hog1 activation, although the underlying mechanism remains unclear.^19^ In addition, we recently demonstrated that acetic acid stress promotes the Pbs2-dependent phosphorylation of Hog1 through the formation of stress granules.^20^ These stress granules function as scaffolding platforms that bring Hog1 and Pbs2 into close proximity, thereby enabling Hog1 phosphorylation without the activation of upstream HOG signaling components, including the MAP3K Ste11. Thus, non-osmotic stresses can activate Hog1 through mechanistically diverse routes, including the engagement of only a subset of HOG pathway components rather than the full upstream-to-downstream cascade.

Hyperosmotic stress, however, represents the most potent and physiologically relevant stimulus for Hog1 activation, and activation of the HOG pathway is essential for yeast survival under hyperosmotic conditions.^2^ This raises an important conceptual question: How does the HOG pathway ensure that robust Hog1 activation is permitted only under hyperosmotic stress conditions, even if individual upstream components become activated under other conditions?

Within the SHO1 branch, the TM mucins Hkr1 and Msb2 are thought to function as partially redundant osmosensors. Simultaneous disruption of *HKR1* and *MSB2* causes pronounced osmosensitivity and abolishes Hog1 activation in response to hyperosmotic stress in an *ssk2/22Δ* background, whereas the deletion of either gene alone has little effect.^11^ Despite their structural similarity, Hkr1 and Msb2 employ distinct signaling mechanisms.^16^^,^^21^ Both proteins associate with the single-pass TM protein Opy2 via interactions between their Hkr1/Msb2 homology (HMH) domains and the cysteine-rich (CR) domain of Opy2.^8^^,^^10^ Opy2, in turn, connects to Ste11 indirectly through the adaptor protein Ste50.^9^^,^^22^ Msb2 also interacts indirectly with Ste20 via the scaffold protein Bem1, thereby promoting Ste11 activation by Ste20.^16^ Hkr1 is similarly assumed to facilitate Ste11 activation by Ste20, potentially through an unidentified adaptor protein.^16^ Consistent with this view, overexpression of constitutively active Hkr1ΔSTR or Msb2ΔSTR induces Hog1 activation even in the absence of hyperosmotic stress, and this activation requires Ste20.^11^ However, whether Hkr1, Msb2, and Opy2 also participate in Pbs2 phosphorylation by activated Ste11 remains unclear.

Another key osmosensor in the SHO1 branch is Sho1, a four-TM-domain protein that interacts with Hkr1, Msb2, and Opy2 through TM-TM interactions to form a multicomponent signaling complex essential for Hog1 activation. Our previous work suggested that Sho1 plays a direct role in mediating Pbs2 phosphorylation by activated Ste11.^8^ Hyperosmotic stress induces structural rearrangements within the Sho1 TM domains and enhances the association between Sho1 and Ste50. Because Sho1 binds directly to Pbs2, this osmotic enhancement of Sho1–Ste50 binding is thought to constitute a permissive step that enables Ste11-mediated Pbs2 phosphorylation by bringing the Sho1–Pbs2 and Ste50–Ste11 complexes into close proximity.

In addition to these upstream regulatory steps, we previously demonstrated that hyperosmotic stress also acts at the level of Hog1 phosphorylation by Pbs2.^18^ Monophosphorylated Pbs2 (Thr518) can efficiently phosphorylate Hog1 only under hyperosmotic stress conditions, and this osmotic enhancement occurs independently of upstream TM osmosensors in the SHO1 branch. Importantly, the absence of this regulation suppresses Hog1 activation driven by basal MAP3K activity and prevents inappropriate pathway activation and crosstalk from the pheromone MAPK pathway, which shares Ste11 as a signaling component.

In this study, we show that hyperosmotic stress regulates the SHO1 branch at three discrete steps: activation of Ste11 by its upstream kinases Ste20/Cla4, phosphorylation of Pbs2 by activated Ste11, and phosphorylation of Hog1 by Pbs2. Together, these findings reveal that the SHO1 branch is governed by multiple osmosensing mechanisms acting in tandem, such that productive signal transmission is gated at multiple steps and permitted only when hyperosmotic stress is present. This multilayered control architecture ensures that the HOG pathway is robustly and specifically activated in response to hyperosmotic stress, while preventing inappropriate activation under non-osmotic conditions.

## Results

### A strategy to dissect the SHO1 branch of the HOG pathway

Although putative TM osmosensors such as Sho1, Hkr1, Msb2, and Opy2 are known to participate in the activation of the SHO1 branch, the precise signaling steps at which they function remain unclear. Previous studies have relied primarily on downstream readouts—such as Hog1 phosphorylation and Hog1-dependent reporter gene expression—to infer the roles of these TM osmosensors. However, to identify the specific steps at which they act, it is necessary to directly examine the phosphorylation states of upstream kinases, including Pbs2 and Ste11. In this study, we sought to identify the specific steps within the SHO1 branch at which TM proteins function by monitoring the phosphorylation of Pbs2 at Thr518.

The Ste11 MAP3K is phosphorylated and activated by the kinases Ste20 and Cla4 (Ste20/Cla4),^13^^,^^15^^,^^23^ enabling it to phosphorylate the Pbs2 MAP2K at Thr518 under hyperosmotic conditions.^18^ In this study, our strategy was to examine the effects of various mutations on Pbs2 phosphorylation in cells expressing either wild-type Ste11 (Ste11-WT) or a constitutively active Ste11-DDD mutant. Ste11-DDD carries phosphomimetic Asp substitutions at all three activating phosphorylation sites (Ser281, Ser285, and Thr286), thereby bypassing the requirement for Ste20/Cla4-mediated Ste11 activation.^13^ Although Ste11-DDD does not fully recapitulate the physiological mode of Ste11 activation, it serves as a useful genetic tool to functionally separate signaling events occurring upstream and downstream of Ste11. This approach allowed us to determine whether the protein or protein domain affected by a given mutation is required for (1) phosphorylation of Ste11 by the upstream kinases Ste20/Cla4 or (2) phosphorylation of Pbs2 by activated Ste11. For simplicity, we refer to these as the pre-Ste11 step (Ste11 activation by Ste20/Cla4) and the post-Ste11 step (Pbs2 phosphorylation by Ste11). As noted in the Introduction, these TM proteins are not required for the third step, namely phosphorylation of Hog1 by activated Pbs2, and this step was therefore not analyzed further here.

### Activated Ste11 MAP3K requires hyperosmotic stress for Pbs2 phosphorylation

In this study, all strains used contained only the SHO1 branch, as the SLN1 branch was inactivated by the deletion of *SSK2* and *SSK22* (hereafter referred to as *ssk2/22Δ*). First, we confirmed that Ste11 phosphorylates Pbs2 at Thr518 in cells exposed to moderate hyperosmotic stress (0.6 M NaCl) using a *ste11Δ ssk2/22Δ* strain that harbors a plasmid expressing wild-type Ste11 (Ste11-WT) at endogenous levels (Figure 1B, lanes 3–4).

Next, we examined the effect of a constitutively active Ste11 mutant, Ste11-DDD, on Pbs2 phosphorylation. Expression of Ste11-DDD at endogenous levels did not induce Pbs2 phosphorylation under unstressed conditions but did so upon exposure to hyperosmotic stress (Figure 1B, lanes 5–6). This result suggests that even activated Ste11 requires hyperosmotic stress to phosphorylate Pbs2. Alternatively, Ste20 and Cla4 might further activate Ste11-DDD through phosphorylation at additional sites, or hyperosmotic stress might activate an unidentified regulator of Ste11.

To test these possibilities, we analyzed Pbs2 phosphorylation in *ste11Δ ssk2/22Δ* cells expressing either STE11-WT or STE11-DDD under conditions in which Ste20 and Cla4 activities were eliminated (*ste20Δ cla4-td*). The *cla4-td* allele is temperature-sensitive and loses kinase activity.^15^^,^^24^ At the restrictive temperature, hyperosmotic stress (0.6 M NaCl) failed to induce Pbs2 phosphorylation by Ste11-WT in the *ste20 cla4-td* mutant (Figure 1C, lanes 1–2). In contrast, Ste11-DDD robustly phosphorylated Pbs2 even in the absence of Ste20 and Cla4 activities (Figure 1C, lanes 3–4). These results indicate that hyperosmotic stress is required both for Ste11 activation and, independently, for Pbs2 phosphorylation by activated Ste11. This osmotic requirement may reflect regulation at the level of signal transmission efficiency, for example, through conformational changes in Ste11, Pbs2, or associated components. Furthermore, we have previously shown that hyperosmotic stress is also necessary for subsequent Hog1 phosphorylation by activated Pbs2.^18^ Thus, hyperosmotic stress regulates at least three distinct signaling steps within the SHO1 branch of the HOG pathway, imposing stepwise control over signal transmission through the Ste11–Pbs2–Hog1 MAPK cascade.

### Hkr1 is the principal TM mucin osmosensor in the SHO1 branch

Of the three signaling steps that we propose to be regulated by hyperosmotic stress, osmotic enhancement of Hog1 phosphorylation by Pbs2 occurs independently of the known TM proteins.^18^ In contrast, the other two steps—the pre-Ste11 and post-Ste11 steps—are likely regulated by TM proteins, namely Sho1, Hkr1, Msb2, and Opy2. However, because it was not previously appreciated that multiple distinct steps within the SHO1 branch are regulated by hyperosmotic stress, these TM proteins have collectively been referred to as “osmosensors,” thereby obscuring the identity of the actual osmosensing machinery.

**Figure 2:**
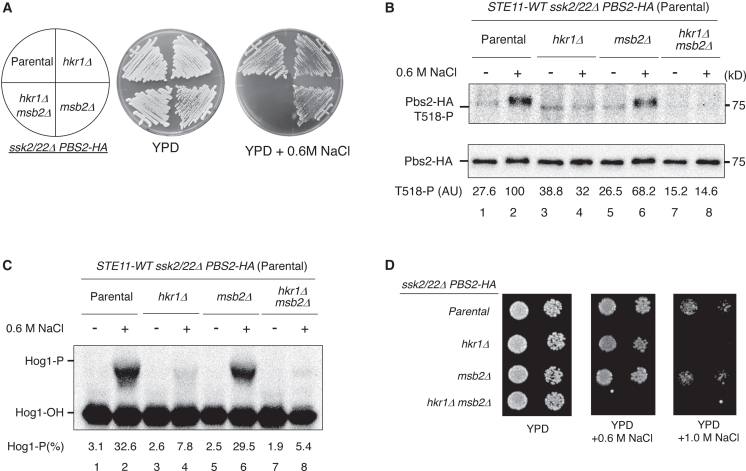
Hkr1, but not Msb2, functions as a TM mucin osmosensor in the SHO1 branch (A) Streak assays for osmotic sensitivity. KT320 (*ste11Δ ssk2/22Δ pbs2*Δ; parental), KT394 (*hkr1Δ*), KT393 (*msb2Δ*), and KT400 (*hkr1Δ msb2Δ*) were streaked onto YPD or YPD +0.6 M NaCl plates and incubated at 30°C. (B) Immunoblot analysis of Pbs2 phosphorylation at Thr518. Cells were treated with 0.6 M NaCl or left untreated for 5 min. Immunoprecipitation and quantification were performed as described in Figure 1B. (C) Phos-tag band-shift analysis of Hog1 phosphorylation. Cells were treated with 0.6 M NaCl or left untreated for 5 min. Phosphorylated (Hog1-P) and unphosphorylated (Hog1-OH) forms of Hog1 are indicated. (D) Spot assays for osmotic sensitivity. Exponentially growing cells were serially diluted and spotted onto YPD or YPD +0.6/1.0 M NaCl plates, incubated at 30°C. Representative immunoblots from at least two independent experiments are shown. Densitometric values are shown for the same representative experiments.

With this issue in mind, we first re-evaluated the regulatory roles of the two TM mucins, Hkr1 and Msb2, in the SHO1 branch. We confirmed that simultaneous deletion of *HKR1* and *MSB2*, but not the deletion of either gene alone, conferred osmosensitivity to *ssk2/22Δ* cells (in which only the SHO1 branch is active) at 0.6 M NaCl (Figure 2A).^11^ We previously reported that Hkr1 and Msb2 activate the HOG pathway through distinct mechanisms despite their genetic redundancy in osmoresistance.^16^ To further investigate their functional differences, we examined the effects of deleting *HKR1* or *MSB2* on Pbs2 phosphorylation. Deletion of *HKR1* severely reduced osmotic induction of Pbs2 phosphorylation at Thr518 (Figure 2B, lanes 3–4). In contrast, the deletion of *MSB2* had only a minor effect on Pbs2 phosphorylation under the same conditions (Figure 2B, lanes 5–6). Notably, the *hkr1Δ msb2Δ* double mutation completely abolished Pbs2 phosphorylation (Figure 2B, lanes 7–8). We also examined Hog1 phosphorylation using a Phos-tag band-shift assay in the same set of mutant strains. Consistent with the Pbs2 phosphorylation patterns, deletion of *HKR1* markedly reduced osmotic induction of Hog1 phosphorylation, whereas the deletion of *MSB2* did not (Figure 2C).

Although these biochemical results initially appeared inconsistent with the growth phenotypes observed at 0.6 M NaCl, closer inspection revealed weak basal phosphorylation of Pbs2 under non-stress conditions in the *hkr1Δ MSB2* mutant (Figure 2B, lane 3), which was completely absent in the *hkr1Δ msb2Δ* mutant (Figure 2B, lane 7). We therefore considered the possibility that this residual Pbs2 activity is sufficient to support cell growth under moderate hyperosmotic stress. To test this idea, we exposed the same set of mutant strains to stronger osmotic stress (1.0 M NaCl). As shown in Figure 2D, *hkr1Δ MSB2* cells grew nearly as well as *HKR1 MSB2* cells at 0.6 M NaCl but failed to grow at 1.0 M NaCl. Together, these results indicate that Hkr1, but not Msb2, is the principal TM mucin functioning as the osmosensor in the SHO1 branch, whereas Msb2 likely contributes to basal Pbs2 activity under non-stress conditions. This functional division is consistent with the role of Msb2 in activating Ste11 in the filamentous growth (FG) pathway.^21^^,^^25^

### Hkr1 regulates both pre- and post-Ste11 steps through distinct mechanisms

**Figure 3:**
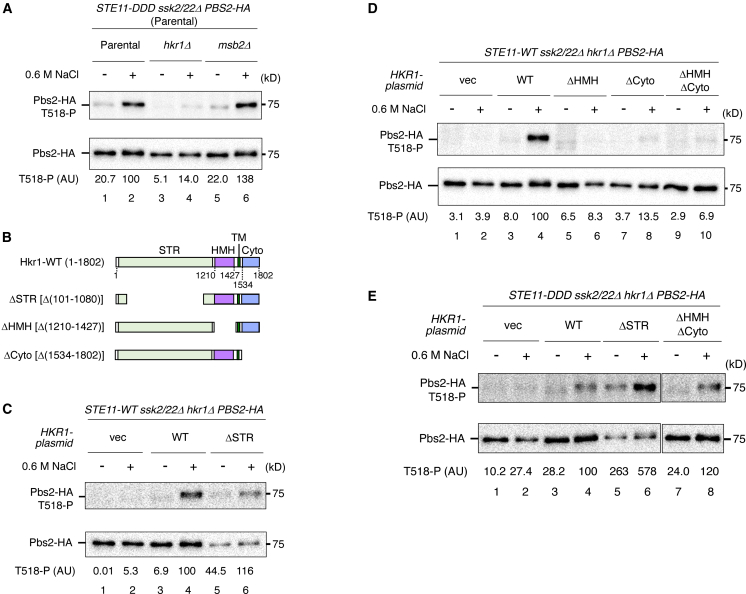
Hkr1 functions in both pre- and post-Ste11 steps through distinct mechanisms (A) Immunoblot analysis of Pbs2 phosphorylation at Thr518. KT320 (parental), KT394 (*hkr1Δ*), and KT393 (*msb2Δ*) were transformed with YCplac22I′-Pbs2-HA and pRS416-STE11-DDD. Immunoprecipitation and quantification were performed as described in Figure 1B. (B) Schematic diagrams of Hkr1-WT and its deletion constructs used in this study. (C) Immunoblot analysis of Pbs2 phosphorylation at Thr518 in KT394 (*hkr1Δ*) co-transformed with YCplac22I′-Pbs2-HA, pRS413-STE11-WT, and either Hkr1-WT, Hkr1-ΔSTR, or vector control. (D) As in (C), except that Hkr1-ΔHMH, Hkr1-ΔCyto, or Hkr1-ΔHMH ΔCyto were used. (E) As in (C), except that pRS413-STE11-DDD was used, and Hkr1-ΔHMH ΔCyto was additionally included. vec, vector; WT, wild type. Representative immunoblots from at least two independent experiments are shown. Densitometric values are shown for the same representative experiments.

To determine whether Hkr1 functions in the pre-Ste11 step, the post-Ste11 step, or both, we examined the effects of *HKR1* deletion, with *MSB2* deletion as a control, on Pbs2 phosphorylation mediated by Ste11-DDD. Deletion of *HKR1* abolished Pbs2 phosphorylation induced by Ste11-DDD at 0.6 M NaCl, whereas the deletion of *MSB2* had no effect (Figure 3A). These results indicate that Hkr1 is required for the post-Ste11 step in the SHO1 branch. However, these data alone do not allow us to determine whether Hkr1 also functions in the pre-Ste11 step.

We therefore examined which functional or structural domains of Hkr1 are required for the pre- and/or post-Ste11 steps. To this end, we constructed expression vectors encoding Hkr1 variants lacking the STR (Ser/Thr-rich), Hkr1/Msb2 homology, or Cyto (cytoplasmic) domains (Figure 3B). Plasmids expressing either wild-type Hkr1 or the deletion derivatives under the control of the *HKR1* promoter were individually introduced into *ssk2/22Δ hkr1Δ* cells, which also carried a plasmid expressing Pbs2-HA. We then monitored Pbs2 phosphorylation at Thr518 mediated by Ste11-WT in the presence or absence of hyperosmotic stress (0.6 M NaCl).

In the absence of Hkr1, no Pbs2 phosphorylation was detected even under hyperosmotic stress (Figure 3C, lanes 1–2), consistent with the results shown in Figure 2B. Deletion of the STR domain did not abolish the osmotic activation of Pbs2 by Ste11-WT (Figure 3C, lanes 5–6). In contrast, deletion of either the HMH or Cyto domain, alone or in combination, completely abolished Pbs2 phosphorylation mediated by Ste11-WT (Figure 3D, lanes 5–10). Notably, the same mutation (Hkr1ΔHMHΔCyto) did not inhibit Pbs2 phosphorylation by Ste11-DDD (Figure 3E, lanes 7–8), indicating that the HMH and Cyto domains are required for the pre-Ste11 step, but not for the post-Ste11 step. Consistent with this domain-specific requirement, deletion of the STR domain enhanced Pbs2 phosphorylation by Ste11-DDD even in the absence of hyperosmotic stress (Figure 3E, lanes 5–6). We previously reported that overexpression of Hkr1ΔSTR induced Hog1 activation under non-stress conditions, although the specific step within the SHO1 branch responsible for this effect could not be determined at that time.^11^ In light of the present results, we now conclude that Hkr1ΔSTR constitutively activates the post-Ste11 step.

We previously showed that the Cyto domain of Hkr1 is essential for Hog1 activation by the constitutively active Hkr1ΔSTR mutant, based on experiments using overexpressed Hkr1ΔSTRΔCyto constructs.^16^ This observation can now be interpreted as follows: Although Hkr1ΔSTR constitutively activates the post-Ste11 step, Ste11 must first be activated for this signaling to occur, and activation of Ste11 requires the Hkr1 Cyto domain. Consequently, overexpressed Hkr1ΔSTRΔCyto is unable to activate Pbs2.

### Sho1 and Ste50 are required for the post-Ste11 step, whereas Opy2 is dispensable

**Figure 4:**
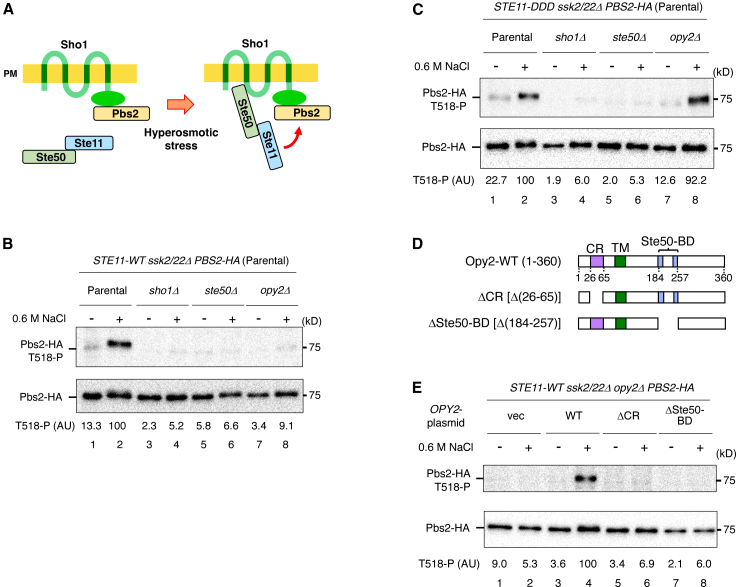
Sho1 and Ste50 are required for the post-Ste11 step, whereas Opy2 is dispensable (A) Schematic model of Pbs2 phosphorylation by activated Ste11, triggered by the stress-induced association of Ste50 and Sho1. (B) Immunoblot analysis of Pbs2 phosphorylation at Thr518. KT320 (parental), KT391-2 (*sho1Δ*), KT404 (*ste50Δ*), and KT392 (*opy2Δ*) were transformed with YCplac22I′-Pbs2-HA and pRS416-STE11-WT. Immunoprecipitation and quantification were performed as described in Figure 1B. (C) As in (B), except that pRS416-STE11-DDD was used. (D) Schematic diagrams of Opy2-WT and its deletion constructs used in this study. (E) Immunoblot analysis of Pbs2 phosphorylation at Thr518 in KT392 (*opy2Δ*) co-transformed with YCplac22I′-Pbs2-HA, pRS413-STE11-WT, and either Opy2-WT, Opy2-ΔCR, Opy2-ΔSte50-BD, or vector control. vec, vector; WT, wild type. Representative immunoblots from at least two independent experiments are shown. Densitometric values are shown for the same representative experiments.

Ste50, which constitutively associates with Ste11, is essential for the Ste11-mediated activation of the SHO1 branch. We previously showed that hyperosmotic stress induces the association of Ste50 with Sho1, whereas Sho1 constitutively binds to Pbs2.^8^ These observations led us to hypothesize that the hyperosmotic stress-induced Sho1–Ste50 interaction promotes the formation of a Ste11–Pbs2 signaling complex, thereby facilitating the phosphorylation of Pbs2 by activated Ste11 (Figure 4A). To test this model, we examined the effects of deleting *STE50*, *SHO1*, or *OPY2* on Pbs2 phosphorylation mediated by either Ste11-WT or Ste11-DDD. Deletion of *STE50*, *SHO1*, or *OPY2* abolished hyperosmotic stress-induced Pbs2 phosphorylation by Ste11-WT (Figure 4B), consistent with previous reports showing that all three genes are required for Hog1 activation through the SHO1 branch. In contrast, Pbs2 phosphorylation by Ste11-DDD was abolished by the deletion of *STE50* or *SHO1*, but not by the deletion of *OPY2* (Figure 4C). These results indicate that Ste50 and Sho1 are required for the post-Ste11 step, whereas Opy2 is dispensable at this step and instead functions specifically in the pre-Ste11 step.

Opy2 contains two protein-interaction domains (Figure 4D): an extracellular CR domain that binds the HMH domains of Hkr1,^8^^,^^10^ and a cytoplasmic Ste50-binding domain (Ste50-BD).^26^ To determine whether Opy2 contributes to the pre-Ste11 step through its interactions with Hkr1 and/or Ste50, we analyzed the effects of deleting either the CR or Ste50-BD domain on Pbs2 phosphorylation mediated by Ste11-WT. These experiments were performed in a *STE11 ssk2/22Δ opy2Δ* background. Deletion of either the CR or Ste50-BD domain abolished hyperosmotic stress-induced Pbs2 phosphorylation at 0.6 M NaCl (Figure 4E). Thus, both Opy2–Hkr1 and Opy2–Ste50 interactions are required for the pre-Ste11 step, supporting a model in which Opy2 facilitates the pre-Ste11 step by tethering Hkr1 to the Ste50–Ste11 complex.

### Osmotically induced association between the Ste50–Ste11 and the Pbs2–Sho1 complexes is required for the post-Ste11 step

**Figure 5:**
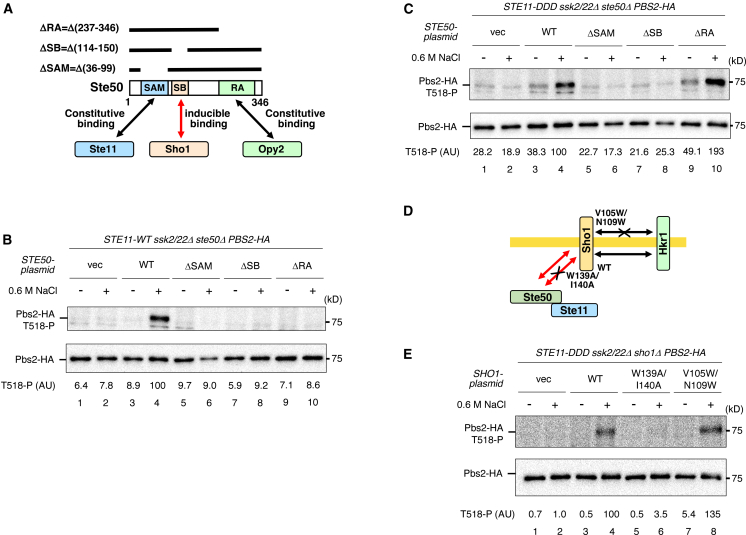
Hyperosmotic stress-inducible interaction between Sho1 and Ste50 is essential for Pbs2 phosphorylation by activated Ste11 (A) Schematic diagram of Ste50 constructs with protein-binding domain deletions. (B) Immunoblot analysis of Pbs2 phosphorylation at Thr518. KT404 (*ste50Δ*) was co-transformed with pRS416-PBS2-HA, pRS413-STE11-WT, and Ste50-WT, Ste50-ΔSAM, Ste50-ΔSB, Ste50-ΔRA, or vector control. Immunoprecipitation and quantification were performed as described in Figure 1B vec, vector; WT, wild type. (C) As in (B), except pRS416-STE11-DDD was used. (D) Schematic diagram of Sho1 mutations that reduce association with Ste50 (*sho1-W139A/I140A*) or Hkr1 (*sho1-V105W/N109W*). (E) Immunoblot analysis of Pbs2 phosphorylation at Thr518. KT391-2 (*sho1Δ*) was co-transformed with YCplac22I′-Pbs2-HA, pRS413-STE11-DDD, and either Sho1-WT, Sho1-W139A/I140A, Sho1-V105W/N109W, or vector control. vec, vector; WT, wild type. Representative immunoblots from at least two independent experiments are shown. Densitometric values are shown for the same representative experiments.

To clarify the role of Ste50 in the post-Ste11 step, we examined the effects of deleting functional domains of Ste50 on Ste11-dependent phosphorylation of Pbs2. Ste50 contains at least three protein-binding domains: a SAM domain that binds Ste11, an SB domain that binds Sho1, and an RA domain that binds Opy2^8^^,^^27^^,^^28^ (Figure 5A). We co-expressed plasmid-borne Ste50-WT or its deletion mutants (or an empty vector) together with either Ste11-WT or Ste11-DDD in *ste11Δ ssk2/22Δ ste50Δ* cells expressing Pbs2-HA. Phosphorylation of Pbs2 at Thr518 was then analyzed in the presence or absence of 0.6 M NaCl. Deletion of the RA domain abolished Pbs2 phosphorylation by Ste11-WT, but not by Ste11-DDD (Figures 5B and 5C, lanes 9–10), consistent with the conclusion that Opy2 is required specifically for the pre-Ste11 step. In contrast, deletion of either the SAM or SB domain eliminated Pbs2 phosphorylation by both Ste11-WT and Ste11-DDD under hyperosmotic conditions (Figures 5B and 5C, lanes 5–8). These results indicate that both the Ste50–Ste11 and Ste50–Sho1 interactions are required for the post-Ste11 step. This conclusion was further supported by the observation that the *sho1*-*W139A I140A* mutation, which inhibits hyperosmotic stress-induced association of Sho1 with Ste50,^8^ abolished Pbs2 phosphorylation by Ste11-DDD under hyperosmotic conditions (Figure 5E, lanes 5–6). Another Sho1 mutant, *sho1-V105W N109W*, which inhibits the TM-mediated interaction between Sho1 and Hkr1, had no inhibitory effect on Ste11-DDD-dependent Pbs2 phosphorylation (Figure 5E, lanes 7–8).

**Figure 6:**
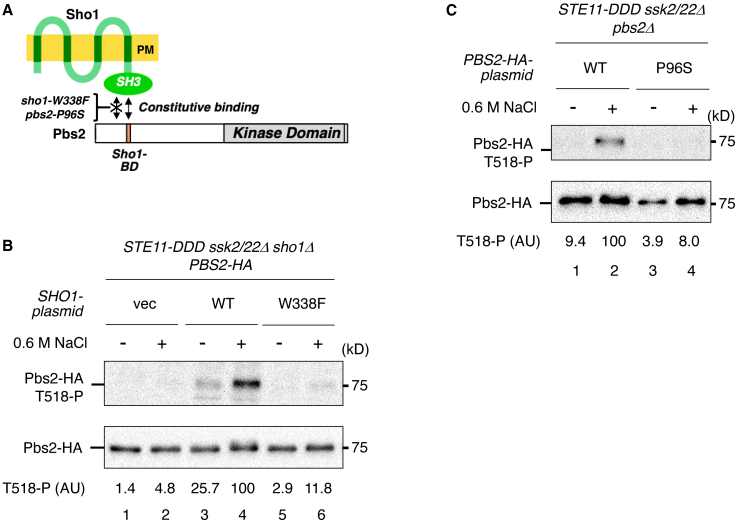
The Sho1–Pbs2 interaction is essential for Pbs2 phosphorylation by activated Ste11 (A) Schematic diagram of the constitutive Sho1–Pbs2 interaction. Mutations that reduce the interaction, *sho1-W338F* and *pbs2-P96S*, are indicated. (B) Immunoblot analysis of Pbs2 phosphorylation at Thr518. KT391-2 (sho1Δ) was co-transformed with YCplac22I′-Pbs2-HA, pRS413-STE11-DDD, and Sho1-WT, Sho1-W338F, or vector control. Immunoprecipitation and quantification were performed as described in Figure 1B vec, vector; WT, wild type. (C) Immunoblot analysis of Pbs2 phosphorylation at Thr518. KT320 (parental) was transformed with pRS413-STE11-DDD and either YCplac22I′-Pbs2-HA or YCplac22I′-Pbs2-P96S-HA. Analysis was performed as described in Figure 1B vec, vector; WT, wild type. Representative immunoblots from at least two independent experiments are shown. Densitometric values are shown for the same representative experiments.

Next, we assessed the importance of the Sho1–Pbs2 interaction in Pbs2 phosphorylation by activated Ste11. The cytoplasmic SH3 domain of Sho1 constitutively binds an SH3-binding motif in Pbs2 (Figure 6A). To disrupt this interaction, we used the *sho1*-*W338F* and *pbs2*-*P96S* mutations, both of which reduce the affinity between Sho1 and Pbs2.^7^^,^^29^ Both mutations abolished Pbs2 phosphorylation by Ste11-DDD under hyperosmotic conditions (Figures 6B and 6C), indicating that Sho1–Pbs2 binding is essential for Pbs2 phosphorylation by activated Ste11 in the post-Ste11 step. Together, these results demonstrate that multiple interactions—Ste11–Ste50, Ste50–Sho1, and Sho1–Pbs2—are required for Pbs2 phosphorylation during the post-Ste11 step.

**Figure 7:**
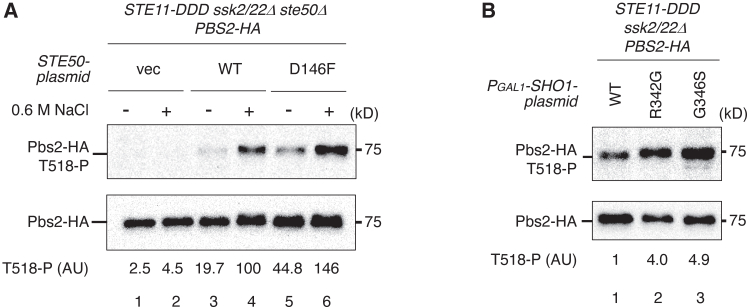
Forced interaction between Ste50 and Sho1 promotes Pbs2 phosphorylation by activated Ste11 in the absence of hyperosmotic stress (A) Immunoblot analysis of Pbs2 phosphorylation at Thr518. KT404 (*ste50Δ*) was co-transformed with YCplac22I′-Pbs2-HA, pRS413-STE11-DDD, and either Ste50-WT, Ste50-D146F, or vector control. Immunoprecipitation and quantification were performed as described in Figure 1B vec, vector; WT, wild type. (B) Immunoblot analysis of Pbs2 phosphorylation at Thr518. KT320 (parental) was co-transformed with YCplac22I′-Pbs2-HA, pRS413-STE11-DDD, and a pRS416-PGAL1 plasmid expressing Sho1-WT, Sho1-R342G, or Sho1-G346S. Cells were induced with 2% galactose for 80 min prior to 0.6 M NaCl treatment for 5 min. Analysis was performed as described in Figure 1B. WT, wild type. Representative immunoblots from at least two independent experiments are shown. Densitometric values are shown for the same representative experiments.

Among these interactions, only the Ste50–Sho1 association is inducible in response to hyperosmotic stress, whereas the others are constitutive. This observation suggests that Pbs2 phosphorylation by activated Ste11 may be triggered by the osmotically induced interaction between Ste50 and Sho1. To test this hypothesis, we examined whether enhanced Ste50–Sho1 binding in the absence of hyperosmotic stress could promote Pbs2 phosphorylation by Ste11-DDD. For this purpose, we used a previously characterized Ste50 mutant, *ste50-D146F*, which exhibits increased affinity for Sho1.^15^ Expression of Ste50-D146F at near-endogenous levels significantly enhanced Pbs2 phosphorylation compared with Ste50-WT in the absence of hyperosmotic stress (Figure 7A). We next tested whether overexpression of high-affinity Sho1 mutants (*sho1-R342G* and *sho1-G346S*), which show increased binding to Ste50,^15^ could similarly enhance Pbs2 phosphorylation in the absence of hyperosmotic stress. Overexpression of Sho1-R342G or Sho1-G346S from the *GAL1* promoter markedly increased Pbs2 phosphorylation compared with Sho1-WT (Figure 7B). Thus, enhanced association between Sho1 and Ste50 is sufficient to trigger the post-Ste11 step, enabling Pbs2 phosphorylation by activated Ste11.

## Discussion

In budding yeast, adaptive responses to hyperosmotic stress are mediated by the HOG signal transduction pathway, whose core consists of a three-kinase MAPK cascade composed of Ste11, Pbs2, and Hog1. However, Ste11 is shared among three distinct MAPK cascades: the SHO1 branch of the HOG pathway (Ste11–Pbs2–Hog1), the pheromone pathway (Ste11–Ste7–Fus3/Kss1), and the FG pathway, which is activated under unfavorable nutritional conditions (Ste11–Ste7–Kss1). This shared use of Ste11 creates an inherent challenge for signaling specificity. Activation of Ste11 has the potential to propagate signals into multiple MAPK pathways. Thus, a central question regarding the HOG pathway is how robust Hog1 activation is restricted specifically to hyperosmotic stress. Several mechanisms that prevent undesirable cross-talk among MAPK pathways have been proposed, including (1) insulation of individual pathways by scaffold proteins, (2) inhibition of competing pathways, and (3) kinetic discrimination.^30^ For instance, modulation of the Ste50–Opy2 association regulates MAPK pathway choice in response to external cues.^9^ Under glucose-rich conditions, Yck1/Yck2-dependent phosphorylation of one of the Ste50-binding domains in Opy2 alters the Ste50–Opy2 interaction, resulting in preferential signal transmission to Hog1 rather than to Fus3 or Kss1. Conversely, phosphorylation of Ste50, triggered by any of the MAP kinases (Hog1, Fus3, or Kss1), reduces the Ste50–Opy2 association, thereby preventing excessive or inappropriate MAPK pathway activation.

**Figure 8:**
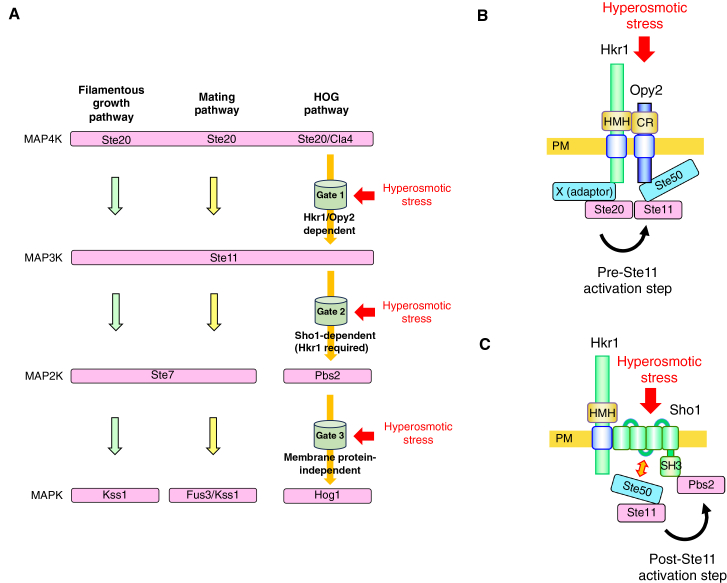
Stepwise, hyperosmotic stress-dependent gating of the HOG pathway through the SHO1 branch (A) Multi-gate control of HOG pathway activation. Hyperosmotic stress regulates three distinct MAPK cascade steps: Gate 1 (Ste20/Cla4–Ste11), Gate 2 (Ste11–Pbs2), and Gate 3 (Pbs2–Hog1). Red arrows indicate regulatory inputs from hyperosmotic stress. Membrane components implicated in each gate are indicated: Gate 1, Hkr1/Opy2; Gate 2, Sho1 with Hkr1 required; Gate 3, membrane protein-independent. Mating and filamentous growth MAPK pathways are shown for comparison. Stepwise gating ensures specific HOG pathway activation despite shared upstream kinases. (B) Hyperosmotic stress-induced Ste11 activation (Gate 1, pre-Ste11 step). Interactions between Hkr1 and Opy2, and between Opy2 and Ste50, bring the Ste20-associated complex close to Ste11, promoting its activation (arrow). An unidentified adaptor (adaptor X) may mediate the Hkr1–Ste20 connection. (C) Hyperosmotic stress-induced Pbs2 phosphorylation by Ste11 (Gate 2, post-Ste11 step). Ste11 is constitutively associated with Ste50, and Pbs2 with Sho1. Upon hyperosmotic stress, Ste50 interacts with Sho1, bringing Ste11 and Pbs2 together for efficient phosphorylation. Hkr1 is required for this step and is positioned adjacent to Sho1, although its precise role remains to be elucidated.

In this manuscript, we show that Hog1 activation is even more stringently restricted to hyperosmotic stress by multiple regulatory gates. In general, an effective way to increase signaling specificity (or equivalently, to reduce noise) is coincidence detection, whereby only events that trigger multiple independent detectors are recorded, as exemplified by scintillation counters for radioactive decay, which register signals only when multiple independent events coincide. Here, we propose that the SHO1 branch of the HOG pathway functions as a coincidence detector equipped with at least three osmotically regulated gates. For simplicity, we refer to these gates, from upstream to downstream, as Gate 1 through Gate 3. We previously identified Gate 3 as an osmotic enhancement of Hog1 phosphorylation by monophosphorylated Pbs2.^18^ Activated Ste11 phosphorylates Pbs2 at only one of its two activating sites, generating monophosphorylated Pbs2 (T518-P). This form of Pbs2 has only weak intrinsic activity toward Hog1 unless further stimulated by hyperosmotic stress. Because hyperosmotic stress does not enhance the Pbs2–Hog1 interaction, osmotic stimulation of Pbs2-dependent Hog1 phosphorylation likely occurs through a distinct mechanism.^18^ Although the precise molecular basis of this regulation remains unresolved, previous work has proposed possible explanations, including the hypothesis that hyperosmotic stress increases the intrinsic susceptibility of Hog1 to phosphorylation by Pbs2. Thus, Gate 3 likely functions to prevent Hog1 activation by irrelevant stimuli in the absence of hyperosmotic stress, and its functional role as a distinct regulatory step is supported by our previous study and incorporated here as a key component of the stepwise gating framework. The other two gates are Gate 1, which regulates the activation of Ste11 by its upstream kinases Ste20 and Cla4, and Gate 2, which regulates the activation of Pbs2 by activated Ste11 (Figure 8A). To focus on the identification of discrete regulatory steps, we analyzed pathway activation under a defined experimental condition using a representative time point corresponding to peak Hog1 activation. Although this approach does not capture the full temporal dynamics or dose dependence of the response, it enables clear discrimination of signal transmission across genetic backgrounds.

Components involved in Gate 1 (the pre-Ste11 step) were identified by mutations that impair the osmotic activation of Hog1 in the presence of Ste11-WT but still allow Hog1 activation when the constitutively active Ste11-DDD allele is expressed. These components include the HMH and Cyto domains of Hkr1, the CR and SB domains of Opy2, and the RA domain of Ste50. The Hkr1-HMH domain binds to the Opy2-CR domain, and the Opy2-SB domain binds to the Ste50-RA domain. Thus, direct interactions between Hkr1 and Opy2, and between Opy2 and Ste50, are specifically required for Gate 1 function. These observations further support our earlier model in which the Hkr1–Opy2 interaction bridges the Ste11 MAP3K, bound to Opy2 via Ste50, to the Ste20 MAP4K (possibly through an unidentified adaptor), thereby promoting Ste11 activation (^16^; Figure 8B). The role of the Hkr1 Cyto domain remains unclear; however, it has been shown to bind the cytoplasmic scaffold protein Ahk1, which also interacts with Sho1, Ste11, and Pbs2.^17^ Ahk1 may therefore serve as an adaptor linking Hkr1 to Ste20, although this possibility remains to be elucidated. Opy2 has been proposed to function as a component of the Sho1 branch of the HOG pathway. Our previous studies^8^^,^^12^ suggested that Opy2 can promote signaling at the level of the Ste11–Pbs2 step under certain conditions, which may appear inconsistent with the present results showing a primary role upstream of Ste11 under hyperosmotic stress. We interpret these findings in a unified framework in which Opy2 does not act at a fixed position within the cascade, but instead facilitates the assembly or stabilization of signaling complexes. In particular, Opy2 interacts with components such as Sho1 and Ste50 and may promote efficient coupling between them. Under hyperosmotic stress, the interaction between Sho1 and Ste50 is likely enhanced in an Opy2-independent manner, thereby reducing the requirement for Opy2 at downstream steps. In contrast, hyperactive Opy2 mutants may compensate for the absence of hyperosmotic stress by enhancing interactions within the signaling complex, in some cases associated with increased affinity for Sho1, thereby mimicking or substituting for stress-induced Sho1–Ste50 association. This could allow signal propagation at the Ste11–Pbs2 step even when upstream activation is bypassed. Thus, rather than defining a fixed step within the pathway, Opy2 modulates the efficiency of signal transmission in a context-dependent manner.

Components involved in Gate 2 (the post-Ste11 step) were defined as those whose deletion inhibits Pbs2 phosphorylation by constitutively active Ste11-DDD. These include the SAM and SB domains of Ste50 (but not the RA domain), the SH3 domain and the Ste50-binding site of Sho1 (but not the Hkr1-binding site), the Sho1-binding site in Pbs2, and the entire Hkr1 protein (but none of its individual domains). The Ste50-SAM domain constitutively binds Ste11, and the Sho1-SH3 domain constitutively binds Pbs2. In contrast, the Ste50-SB domain binds inducibly to the Ste50-binding site of Sho1 upon hyperosmotic stress. Thus, Ste11 and Pbs2 are brought into close proximity upon hyperosmotic stress (Figure 8C). An additional layer of complexity arises from the differential phosphorylation of Pbs2 by upstream MAP3Ks. Ste11 primarily phosphorylates Pbs2 at T518, whereas Ssk2/22 can phosphorylate both S514 and T518. Although the mechanistic basis for this difference remains unclear, it may reflect distinct regulatory architectures in the SHO1 and SLN1 branches. In the SHO1 branch, our results indicate that signal transmission from Ste11 to Pbs2 is regulated at Gate 2, which functions as a checkpoint for pathway activation. Together with Gate 3, this stepwise regulation likely contributes to signaling specificity by preventing inappropriate activation and limiting crosstalk with other MAPK pathways.

Because both deletion of *SHO1* and the SHO1 SH3-domain W338F mutation abolish Ste11-DDD-mediated Pbs2 phosphorylation, Sho1 is clearly required for Gate 2. These data alone are insufficient to determine whether Sho1 also functions in Gate 1. However, the requirement of Sho1 for hyperosmotic stress-induced activation of the Ste11-Ste7-Kss1 MAPK cascade in *pbs2Δ* or *hog1Δ* mutants^31^ implies that Sho1 participates in the activation of Ste11 by upstream kinases, that is, in Gate 1.

Hkr1 and Msb2 have been proposed to function as redundant osmosensors based on the strong osmosensitivity of the double mutant. However, our analysis at the level of Pbs2 phosphorylation reveals a functional distinction between these proteins. Hkr1 plays a dominant role in mediating pathway activation in response to hyperosmotic stress, whereas Msb2 contributes primarily to basal signaling activity. Because phosphorylation assays capture acute signaling events, whereas growth reflects long-term adaptation to hyperosmotic stress, the relationship between signaling intensity and growth outcomes may not be strictly linear. Sho1, in addition to its role as a scaffold, may also contribute to stress sensing, as it undergoes stress-dependent structural changes and can promote Hog1 activation under certain conditions. Together, these observations support a model in which osmosensing and signal transmission are distributed across multiple components, with Hkr1 as the principal trigger, Msb2 supporting basal activity, and Sho1 functioning both as a stress-responsive element and as a structural platform. Importantly, Gate 1 does not represent a single uniform mechanism but instead integrates multiple inputs that regulate the initiation of signaling.

Complete deletion of Hkr1 inhibits Gate 2. However, we could not identify any individual domain that is required for Gate 2. It is possible that two or more domains in Hkr1 are redundant for its function in Gate 2. Moreover, the deletion of the STR (Ser/Thr-rich) domain of Hkr1 actually enhances Gate 2 activity. Indeed, overexpression of Hkr1ΔSTR strongly activates Hog1 even in the absence of hyperosmotic stress. Thus, Hkr1 appears to exert both positive and negative regulatory effects on Gate 2, which are likely distinct from its role in Gate 1. Although our current analyses are insufficient to fully resolve the functions of Sho1 and Hkr1, we propose the following hypotheses based on the present and previous findings.

Sho1 contains both homo-dimerization and homo-trimerization interfaces within its four-TM domain.^8^ Through repeated dimerization and trimerization, Sho1 can form a large planar multimer within the membrane. Importantly, the dimer interface binds Opy2, whereas the trimer interface binds Hkr1. Thus, the Sho1 multimer may provide a TM platform analogous to a floating pier, on which multiple Hkr1 and Opy2 molecules congregate. The resulting high local concentrations of Hkr1 and Opy2 would enhance the efficiency of Gate 1 signaling.

Hkr1 can associate with Sho1 either directly through their TM domains or indirectly via Opy2.^8^^,^^11^ In either case, juxtaposition of Hkr1 and Sho1 would bring the Gate 1 complex into proximity with the Gate 2 complex, thereby facilitating efficient transfer of activated Ste11 from Gate 1 to Gate 2. The HMH domain of Hkr1 is a mucin-like, Ser/Thr-rich region that is highly glycosylated and likely highly hydrated under low-ionic-strength conditions. The resulting bulkiness of this domain may inhibit interactions between Hkr1 and Opy2 and/or Sho1. At a critical osmolarity, certain hydrogel-forming polymers undergo a rapid transition from a hydrated to a dehydrated state, accompanied by a dramatic reduction in volume.^32^ A similar volume transition in the mucin-like HMH domain could permit interactions among Hkr1, Opy2, and Sho1. If such a mechanism operates *in vivo*, it may underlie osmosensing by Hkr1. These observations suggest that the three-gate architecture defined here primarily applies to osmotic signaling in the SHO1 branch, whereas non-osmotic stresses may activate Hog1 by engaging only a subset of these regulatory steps or without requiring additional activation of upstream components.

In summary, we have shown that the SHO1 branch of the HOG pathway is regulated by hyperosmotic stress through three consecutive, mechanistically distinct gates, conferring exquisite specificity for hyperosmotic conditions. We further propose that these three gates monitor distinct aspects of hyperosmotic stress: Gate 1 senses extracellular conditions via the mucin-like TM protein Hkr1, Gate 2 monitors TM conditions via the four-TM protein Sho1, and Gate 3 monitors intracellular conditions.

The yeast strains and plasmids used in this study are listed in Tables 1 and 2, respectively.

**Table 1:** Yeast strains used in this study

Strain	Genotype	Source
KT320	*MAT***a***ura3 leu2 trp1 his3 ssk2::hisG ssk22::hisG ste11::kanMX6 pbs2:: natMX4*	This study
KT391-2	*MAT***a***ura3 leu2 trp1 his3 ssk2::hisG ssk22::hisG ste11::kanMX6 pbs2:: natMX4 sho1::hphMX4*	This study
KT392	*MAT***a***ura3 leu2 trp1 his3 ssk2::hisG ssk22::hisG ste11::kanMX6 pbs2:: natMX4 opy2::hphMX4*	This study
KT393	*MAT***a***ura3 leu2 trp1 his3 ssk2::hisG ssk22::hisG ste11::kanMX6 pbs2:: natMX4 msb2::hphMX4*	This study
KT394	*MAT***a***ura3 leu2 trp1 his3 ssk2::hisG ssk22::hisG ste11::kanMX6 pbs2:: natMX4 hkr1::hphMX4*	This study
KT396	*MAT*α *leu2 trp1 his3 ssk2::LEU2 ssk22::LEU2 ste11::hphMX4 ste20::kanMX6 cla4::natMX4 URA3::cla4-75-td*	This study
KT400	*MAT***a***ura3 leu2 trp1 his3 ssk2::hisG ssk22::hisG ste11::kanMX6 pbs2:: natMX4 hkr1::hphMX4 msb2:: HIS3MX4*	This study
KT404	*MAT***a***ura3 leu2 trp1 his3 ssk2::hisG ssk22::hisG ste11::kanMX6 pbs2:: natMX4 ste50::hphMX4*	This study

**Table 2:** Plasmids used in this study

Plasmid name	Backbone	Insert	Promoter	Tag	Description	Source
pRS413	pRS413	–	–	–	Single-copy vector	Stratagene
pRS414	pRS414	–	–	–	Single-copy vector	Stratagene
pRS416	pRS416	–	–	–	Single-copy vector	Stratagene
pRS426	pRS426	–	–	–	High-copy vector	Stratagene
YCplac22I	YCplac22I	–	–	–	Single-copy vector	“Gietz & Sugino^34^”
pRS413-Ste11	pRS413	*STE11*	PSTE11	–	Wild-type Ste11 expression	This study
pRS416-Ste11	pRS416	*STE11*	PSTE11	–	Wild-type Ste11 expression	This study
pRS413-Ste11-DDD	pRS413	*STE11-DDD*	PSTE11	–	Constitutively active Ste11	This study
pRS416-Ste11-DDD	pRS416	*STE11-DDD*	PSTE11	–	Constitutively active Ste11	This study
pRS414-Ste50	pRS414	*STE50*	PSTE50	–	Wild-type Ste50 expression	“Tatebayashi et al.^8^”
pRS416-Ste50	pRS416	STE50	PSTE50	–	Wild-type Ste50 expression	“Tatebayashi et al.^8^”
Ste50 mutant plasmids	pRS414/pRS416	*STE50* variants	PSTE50	–	Domain deletion mutants (ΔSAM, ΔSB, ΔRA)	“Tatebayashi et al.^8^”
pRS416-Sho1	pRS416	*SHO1*	PSHO1	–	Wild-type Sho1 expression	“Tatebayashi et al.^8^”
Sho1 mutant plasmids	pRS416	*SHO1* variants	PSHO1	–	Interaction-defective mutants (e.g., W139A/I140A,V105W/N109W, W338F)	“Tatebayashi et al.^8^”
pRS416-PGAL1-Sho1-R342G	pRS416	*SHO1-R342G*	PGAL1	–	Inducible mutant expression	“Tatebayashi et al.^15^”
pRS416-PGAL1-Sho1-G346S	pRS416	*SHO1-G346S*	PGAL1	–	Inducible mutant expression	“Tatebayashi et al.^15^”
YCplac22I′-Pbs2	YCplac22I′	*PBS2*	PPBS2	–	Wild-type Pbs2 expression	“Tatebayashi et al.^33^”
YCplac22I-Pbs2-HA	YCplac22I′	*PBS2*	PPBS2	HA	C-terminally HA-tagged Pbs2	“Tatebayashi et al.^18^”
YCplac22I-Pbs2 mutant plasmids	YCplac22I′	*PBS2* variants	PPBS2	HA	Mutant derivatives (e.g., P96S)	“Tatebayashi et al.^33^”
pRS416-PBS2-HA	pRS416	*PBS2*	PPBS2	HA	HA-tagged Pbs2 (vector-converted)	“Tatebayashi et al.^18^”
pRS416-Hkr1	pRS416	*HKR1*	PHKR1	–	Wild-type Hkr1 expression	“Tatebayashi et al.^11^”
Hkr1 mutant plasmids	pRS416	*HKR1* variants	PHKR1	–	Deletion mutants (e.g., ΔSTR, ΔHMH, ΔCyto)	“Tatebayashi et al.^11^”
pRS416-Opy2	pRS416	*OPY2*	POPY2	–	Wild-type Opy2 expression	“Yamamoto et al.^9^”
Opy2 mutant plasmids	pRS416	*OPY2* variants	POPY2	–	Deletion mutants (ΔCR, ΔSte50-BD, ΔRA)	“Tatebayashi et al.^8^”, This study

Italicized entries indicate gene names.

### Limitations of the study

This study has several limitations. First, our conclusions are based on immunoblot analyses of Pbs2 phosphorylation at a limited number of time points and osmotic conditions, and therefore do not capture the full dynamics of pathway activation. Second, the use of the constitutively active Ste11-DDD allele, while useful for dissecting regulatory steps, may not fully reflect physiological signaling conditions. Third, although Hkr1 is implicated in multiple steps, its precise molecular role remains to be elucidated. Finally, additional regulatory components or context-dependent mechanisms may contribute to pathway control but were not examined here.

## Resource availability

### Lead contact

Further information and requests for resources and reagents should be directed to and will be fulfilled by the lead contact, Kazuo Tatebayashi (tategone@ims.u-tokyo.ac.jp).

### Materials availability

All unique reagents generated in this study are available from the lead contact upon reasonable request.

### Data and code availability

• Data: All data supporting the findings of this study are available from the lead contact upon reasonable request.
• Code: This paper does not report original code.
• Additional Information: No additional information is reported in this paper.

## Acknowledgments

This work was supported in part by 10.13039/501100001691JSPS Grants-in-Aid for Scientific Research (10.13039/501100001691KAKENHI) grant no. 25K09557 and by a grant from the 10.13039/100007802Institute for Fermentation, Osaka (10.13039/100007802IFO) to K.T.

## Author contributions

Conceptualization, K.T. and H.S.; methodology, K.T.; investigation, K.T.; writing – original draft, K.T. and H.S.; writing – review and editing, K.T. and H.S.; funding acquisition, K.T.; resources, K.T. and H.S.; supervision, K.T. and H.S.

## Declaration of interests

The authors declare no competing interests.

## Declaration of generative AI and AI-assisted technologies in the writing process

During the preparation of this work, the authors used ChatGPT-5.2 (OpenAI) to assist with language editing and improvement of clarity. After using this tool, the authors reviewed and edited the content as needed and take full responsibility for the content of the published article.

## STAR★Methods

### Key resources table

**Table:** 

REAGENT or RESOURCE	SOURCE	IDENTIFIER
**Antibodies**		

Anti-Pbs2 phospho-T518 antibody	Scrum Inc.	Custom antibody; (*Tatebayashi*et al.^1^)
Anti-HA antibody (3F10)	Roche	Cat# 11867423001; RRID: AB_390918
Anti-HA antibody (F-7)	Santa Cruz Biotechnology	Cat# sc-7392; RRID: AB_627808
Anti-Hog1 antibody (yC20)	Santa Cruz Biotechnology	Cat# sc-6815; RRID: AB_631392
Anti-mouse IgG-HRP	Cytiva	Cat# NA931V; RRID: AB_772210
Anti-rabbit IgG-HRP	Cytiva	Cat# NA934V; RRID: AB_772206
Anti-goat IgG-HRP	Promega	Cat# V805A; RRID: AB_10015289

**Bacterial and virus strains**		

Escherichia coli DH5α competent cells	Takara Bio	Cat# 9057

**Chemicals, peptides, and recombinant proteins**		

Phos-tag Acrylamide (AAL-107)	Fujifilm Wako	Cat# 300-93521
Dynabeads Protein G	Thermo Fisher Scientific	10004D

**Experimental models: Organisms/strains**		

*Saccharomyces cerevisiae* strains S288C derivatives	This study	See Table 1

**Oligonucleotides**		

Oligonucleotide: PBS2 ΔF: TATATTCACGTGCC TGTTTGCTTTTATTTGGATATTAACGCGGATC CCCGGGTTAATTAA (gene disruption)	This study	N/A
Oligonucleotide: PBS2 ΔR: TATATTCACGTGCC TGTTTGCTTTTATTTGGATATTAACGGA ATTCGAGCTCGTTTAAAC (gene disruption)	This study	N/A
Oligonucleotide: HKR1 ΔF: ACTGACGCAGTCAACA GTCAGAAACAGAGAATGTATAAAGCG GATCCCCGGGTTAATTAA (gene disruption)	This study	N/A
Oligonucleotide: HKR1 ΔR: TTCCAGCAGCG TTTATTGCACAGTCAATAATCATTTTTC GGAATTCGAGCTCGTTTAAAC (gene disruption)	This study	N/A
Oligonucleotide: MSB2 ΔF: TTTATTGACTTTTCA TTAGGCTTCCTAATTATACCCATCTCGGAT CCCCGGGTTAATTAA (gene disruption)	This study	N/A
Oligonucleotide: MSB2 ΔR: AGGTTATGCAA GCGGAGAAAGTCTCTGCAGACATTGCTATGAATTC GAGCTCGTTTAAAC (gene disruption)	This study	N/A
Oligonucleotide: OPY2 ΔF: TCAAACTGGTTAC GTTCGTTTTCTGAAAATCAAACAAAAACGGAT CCCCGGGTTAATTAA (gene disruption)	This study	N/A
Oligonucleotide: OPY2 ΔR: GATATAATATTTTCC CCGGGATTGCAGAATACTGACACGCGAATT CGAGCTCGTTTAAAC (gene disruption)	This study	N/A

**Recombinant DNA**		

YCplac22I′-Pbs2 and derivatives (Table 2)	*Tatebayashi*et al.^33^	N/A
YCplac22I′-Pbs2-HA and derivatives (Table 2)	*Tatebayashi*et al.^33^	N/A
pRS413-Ste11-DDD (Table 2)	This study	N/A
pRS414-Ste50 and derivatives (Table 2)	*Tatebayashi*et al.^8^	N/A
pRS416-Ste50 and derivatives (Table 2)	*Tatebayashi*et al.^8^	N/A
pRS416-Sho1 and derivatives (Table 2)	*Tatebayashi*et al.^8^	N/A
pRS416-PGAL1-Sho1-R342G/G346S (Table 2)	*Tatebayashi*et al.^15^	N/A
pRS416-Hkr1 and derivatives (Table 2)	*Tatebayashi*et al.^11^	N/A
pRS416-Opy2 and derivatives (Table 2)	*Tatebayashi* et al.*,*^8^^,^^9^ This study	N/A

**Software and algorithms**		

Image Lab version 6.1.0	Bio-Rad	N/A
Microsoft Excel for Mac version 16.107.2	Microsoft	N/A

### Experimental model and study participant details

#### Yeast strains

All experiments were performed using Saccharomyces cerevisiae strains derived from the S288C genetic background. The strains used in this study are listed in Table 1. Cells were grown at 30°C in standard yeast extract peptone dextrose (YPD) medium unless otherwise specified. For selection and maintenance of plasmids, synthetic complete (SC) medium lacking appropriate amino acids was used. Gene disruption strains were constructed using PCR-based homologous recombination. The oligonucleotides used for gene disruption of *PBS2*, *HKR1*, *MSB2*, and *OPY2* are provided in the key resources table.

This study did not involve human participants, animals, primary cell cultures, or established cell lines. Therefore, sex- and gender-related variables, cell line authentication, mycoplasma testing, and institutional approval for animal or human studies are not applicable.

### Method details

#### Media and reagents

Yeast cells were grown in rich medium (YPD) or synthetic media as indicated. YPD medium consisted of 1% (w/v) yeast extract, 2% (w/v) tryptone, and 2% (w/v) glucose. Synthetic complete (SC) medium contained 0.67% (w/v) yeast nitrogen base and 2% (w/v) glucose supplemented with appropriate amino acids. CAD medium consisted of 0.67% (w/v) yeast nitrogen base, 2% (w/v) glucose, 0.5% (w/v) casamino acids, and supplements as required (20 μg mL^−1^ uracil and 40 μg mL^−1^ tryptophan). SRaf medium contained 0.67% (w/v) yeast nitrogen base and 2% (w/v) raffinose with appropriate supplements. Phos-tag acrylamide AAL-107 was used for phosphorylation-dependent mobility shift analyses. Other reagents were obtained from standard commercial sources (e.g., Sigma-Aldrich, Nacalai Tesque, and FUJIFILM Wako Pure Chemical).

#### Buffers

Buffer A consisted of 50 mM Tris-HCl (pH 7.5), 15 mM EDTA, 15 mM EGTA, 2 mM dithiothreitol (DTT), 1 mM phenylmethylsulfonyl fluoride (PMSF), 1 mM benzamidine, 5 μg mL^−1^ leupeptin, 150 mM NaCl, and 0.2% (v/v) Triton X-100. Buffer P consisted of 50 mM Tris-HCl (pH 7.5), 2 mM DTT, 1 mM PMSF, 1 mM benzamidine, and 5 μg mL^−1^ leupeptin. TBS (Tris-buffered saline) contained 25 mM Tris-HCl (pH 7.4), 137 mM NaCl, and 2.68 mM KCl. SDS sample buffer (1×) contained 50 mM Tris-HCl (pH 6.8), 2% SDS, 0.01% bromophenol blue, 10% (v/v) glycerol, and 700 mM 2-mercaptoethanol.

#### Plasmid constructs

All plasmids used in this study are listed in Table 2. Most constructs, including mutant derivatives, were generated in previous studies (see Table 2 for details and references). No new oligonucleotides were designed for plasmid construction in this work. Briefly, *STE11*, *STE50*, *SHO1*, *PBS2, HKR1*, and *OPY2* expression plasmids were constructed using genomic DNA fragments expressed under their respective native promoters or, where indicated, the *GAL1* promoter.

#### Yeast culture and hyperosmotic stress treatment

Cells were grown at 30°C in YPD or appropriate selective media. Overnight cultures were diluted to an OD_600_ of 0.25 in fresh YPD and cultured to mid-log phase (OD_600_ = 0.6–0.8) with vigorous aeration. For hyperosmotic stress treatment, cells were harvested by centrifugation at 2,000 × g for 3 min and resuspended in pre-warmed YPD containing the indicated concentrations of NaCl. After incubation, cells were collected by centrifugation and washed with ice-cold Buffer P. Cell pellets were immediately frozen in liquid nitrogen and stored at −80°C until use.

#### Cell extract preparation

Frozen cell pellets were resuspended in Buffer A and disrupted with glass beads at 4°C. Lysates were clarified by centrifugation at 9,200 × g for 10 min at 4°C to remove cell debris. The resulting supernatants were used immediately or stored at −80°C.

#### Immunoprecipitation

Pbs2-HA and its derivatives were immunoprecipitated using Protein G magnetic beads according to the manufacturer’s instructions with minor modifications. Briefly, beads were incubated with anti-HA antibody (3F10; Roche) and then with cell extracts. After washing, bound proteins were eluted by boiling in SDS sample buffer and subjected to SDS-PAGE.

#### Immunoblotting

Proteins were separated by SDS-PAGE and transferred to PVDF or nitrocellulose membranes using a Trans-Blot Turbo system (Bio-Rad) according to the manufacturer’s protocol. Membranes were probed with appropriate primary and HRP-conjugated secondary antibodies. Signals were detected using enhanced chemiluminescence and imaged with a ChemiDoc XRS Plus system (Bio-Rad). Band intensities were quantified using Image Lab software (version 6.1.0, Bio-Rad).

#### Phos-tag SDS-PAGE

Phos-tag SDS-PAGE was performed to resolve phosphorylated protein species. Gels contained Phos-tag acrylamide and Zn^2+^ as described previously. Following electrophoresis, gels were treated with EDTA-containing transfer buffer to remove Zn^2+^ prior to protein transfer.

#### Phospho-Hog1 band-shift assay

Cell lysates were prepared in SDS sample buffer using glass bead disruption. Samples were separated by Phos-tag SDS-PAGE and transferred to PVDF membranes. Hog1 was detected by immunoblotting. The mobility shift of Hog1 was used to distinguish phosphorylated and unphosphorylated forms.

### Quantification and statistical analysis

#### Densitometric quantification of immunoblots

Signal intensities for all immunoblot experiments were quantified by densitometric analysis using Image Lab software 6.1.0 (Bio-Rad Laboratories, Hercules, CA, USA). For the quantification of Pbs2 phosphorylation, the signal intensity of phosphorylated Pbs2 was normalized to the total amount of Pbs2 (detected via the HA-tag). For each independent experiment, the relative phosphorylation level was calculated as a percentage, with the value of the corresponding control strain (e.g., the wild-type for each specific gene—*HKR1, OPY2, SHO1, PBS2, or STE50*—or the parental strain) under hyperosmotic stress conditions set as 100%. In the case of Phos-tag SDS-PAGE analysis, the phosphorylation level of Hog1 was determined by calculating the ratio of the intensity of the slow-migrating band (phosphorylated Hog1) to the total intensity of both the slow- and fast-migrating bands. Numerical labeling of these quantified values has been integrated into the respective figure panels to ensure a consistent and clear presentation of the data.

#### Statistical presentation and reproducibility

As noted in the study design, these experiments were primarily focused on characterizing step-specific signal transmission through representative immunoblot patterns. Therefore, rather than performing formal statistical hypothesis testing (e.g., *p*-value calculation), which may not be appropriate for the specific nature of these biochemical assays, we have provided consistent densitometric quantification to objectively support the observed trends. All presented blots are representative of at least two independent experiments that yielded reproducible results.

## Supplemental information

Data S1. Uncropped western blot images
