# Supplementary material for: Stepwise multi-gate control of the HOG MAPK pathway under hyperosmotic stress
Source: iScience. 2026 May 28;29(6):116132. doi: 10.1016/j.isci.2026.116132 (PMC13233613; doi:10.1016/j.isci.2026.116132)

iScience, Volume *29*

Supplemental information

Stepwise multi-gate control

# of the HOG MAPK

pathway under hyperosmotic stress

Kazuo Tatebayashi and Haruo Saito

Data S1. Uncropped western blot images.

Figure 1B

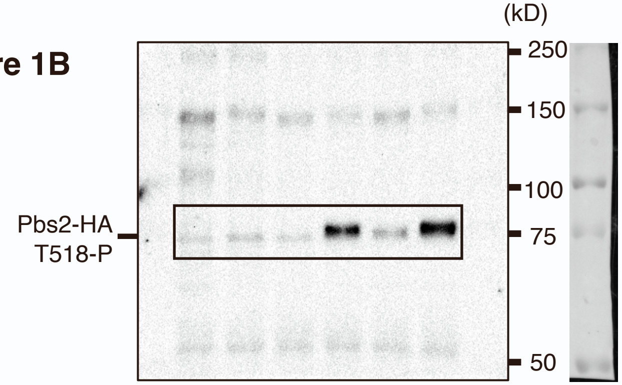

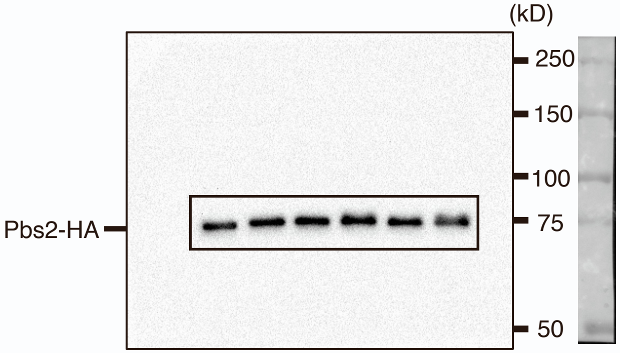

Figure 1C

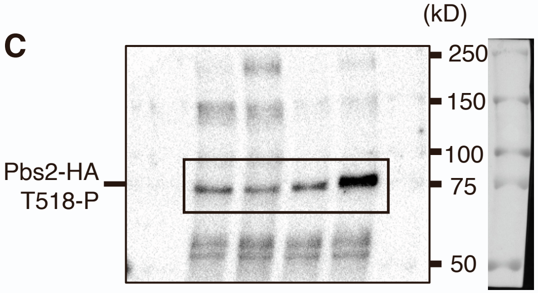

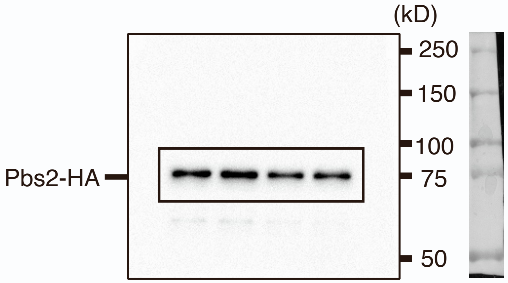

Figure 2B

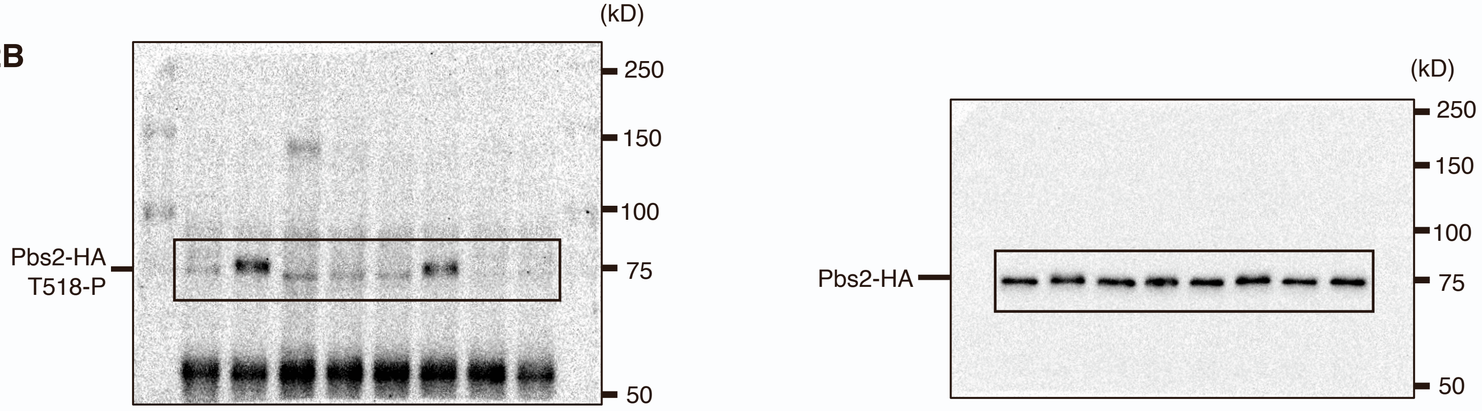

Figure 2C

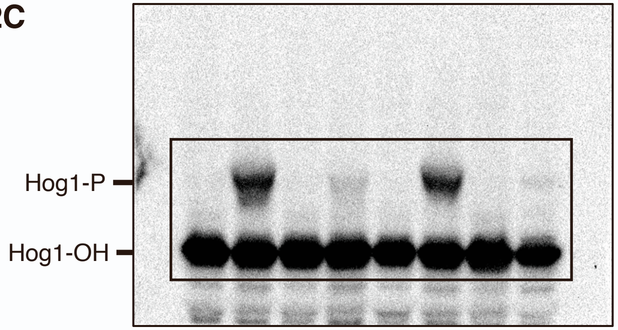

Figure 3A

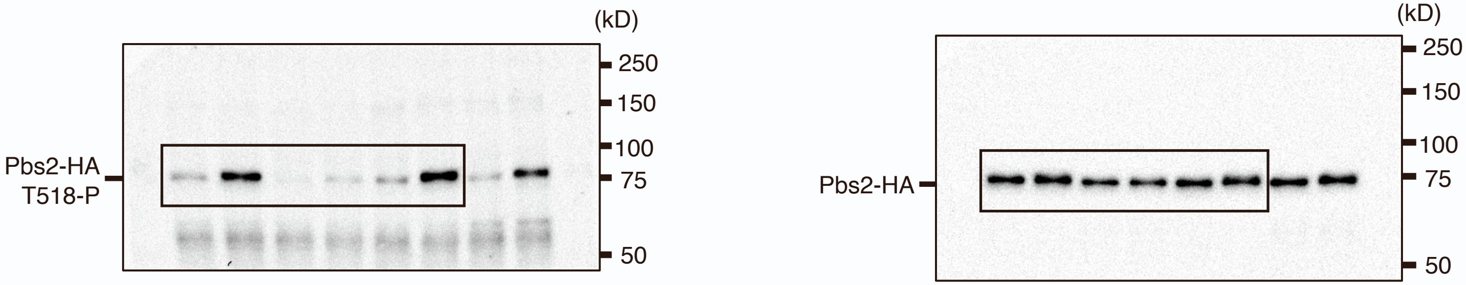

Figure 3C

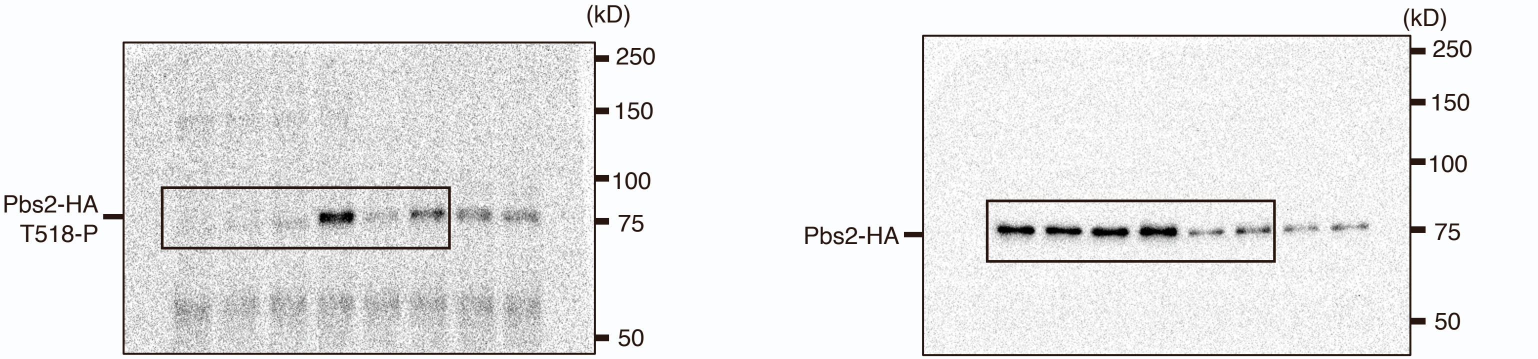

Figure 3D

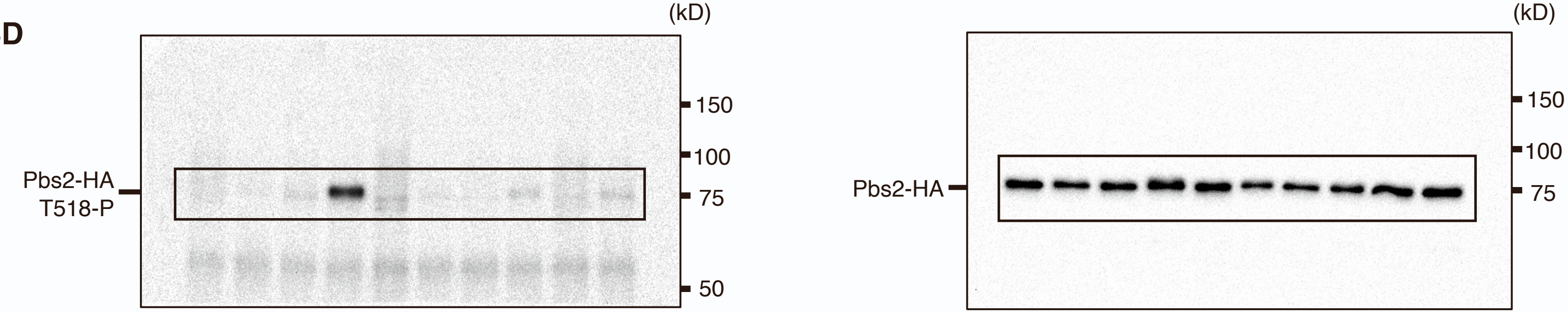

Figure 3E

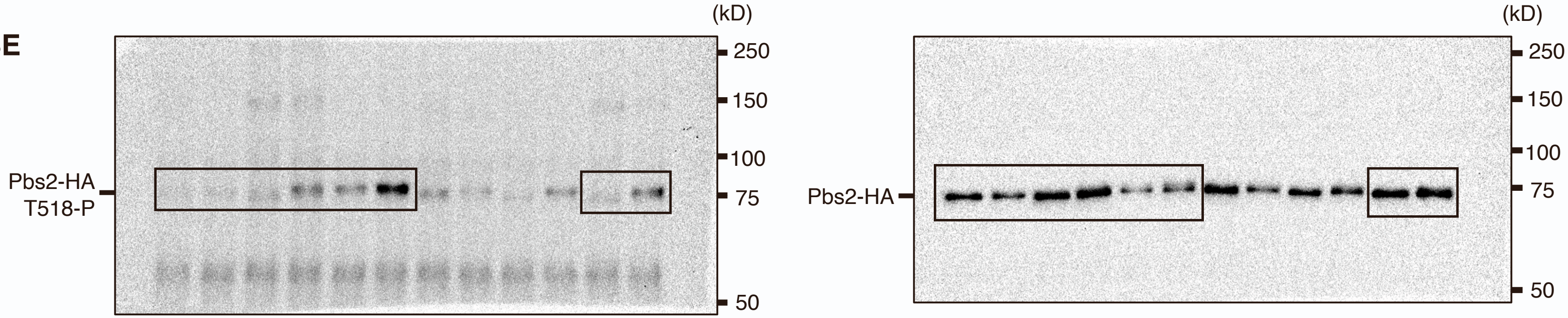

Figure 4B

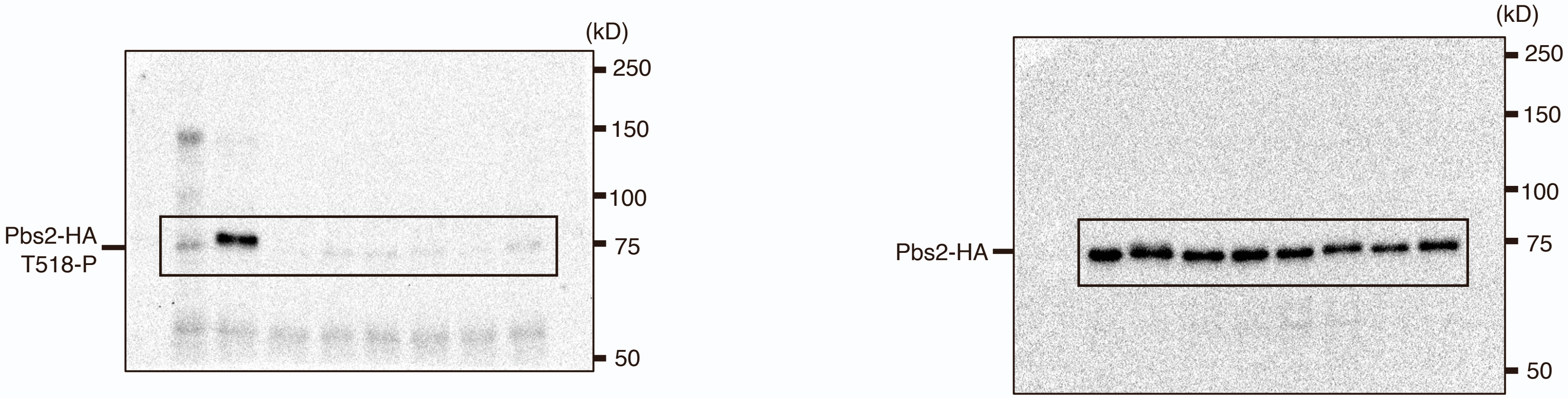

Figure 4C

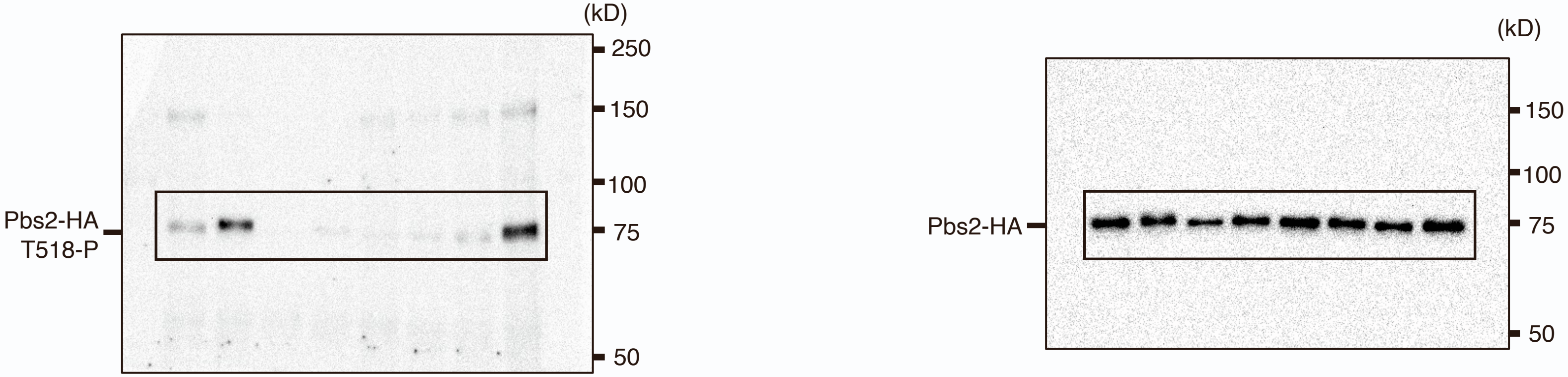

Figure 4E

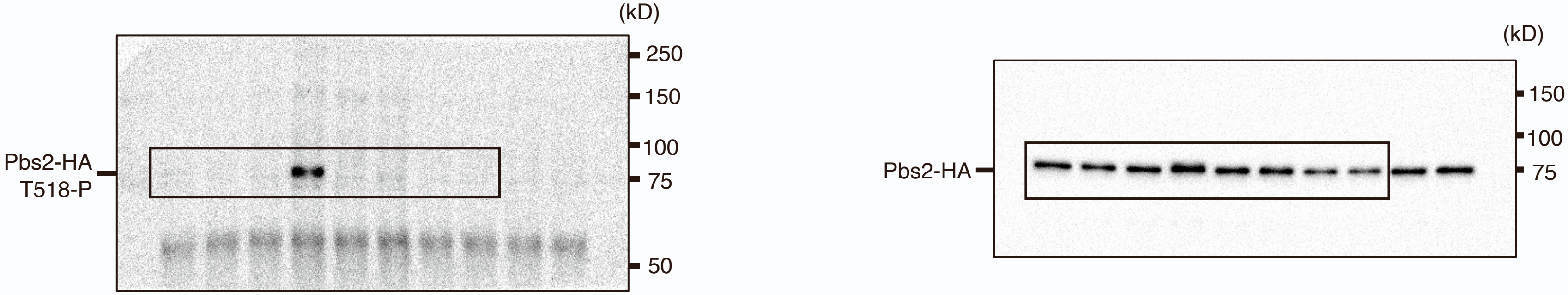

Figure 5B

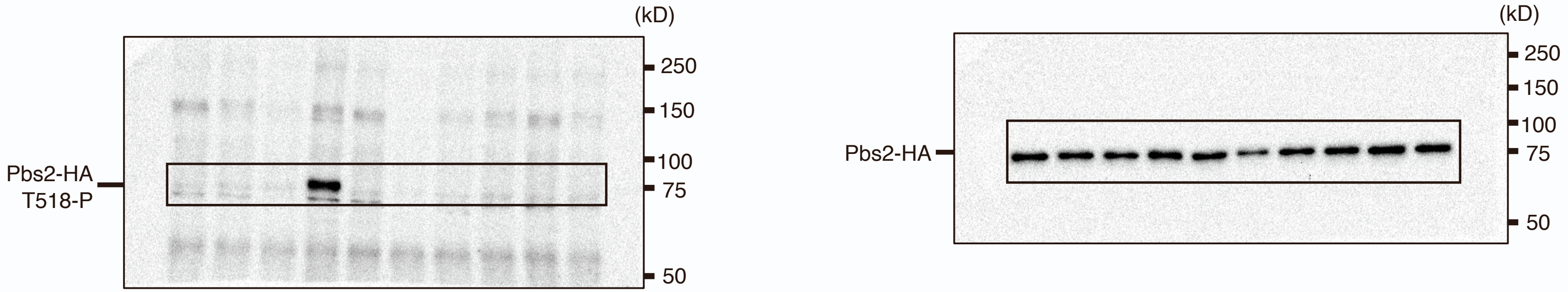

Figure 5C

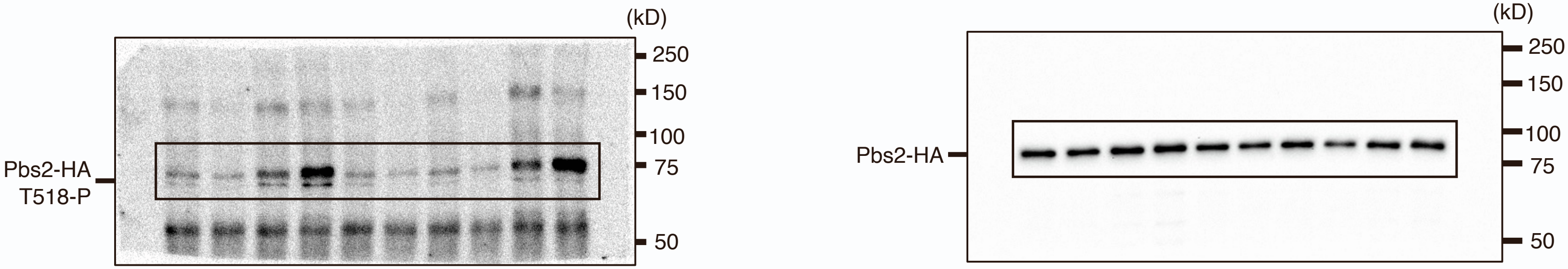

Figure 5E

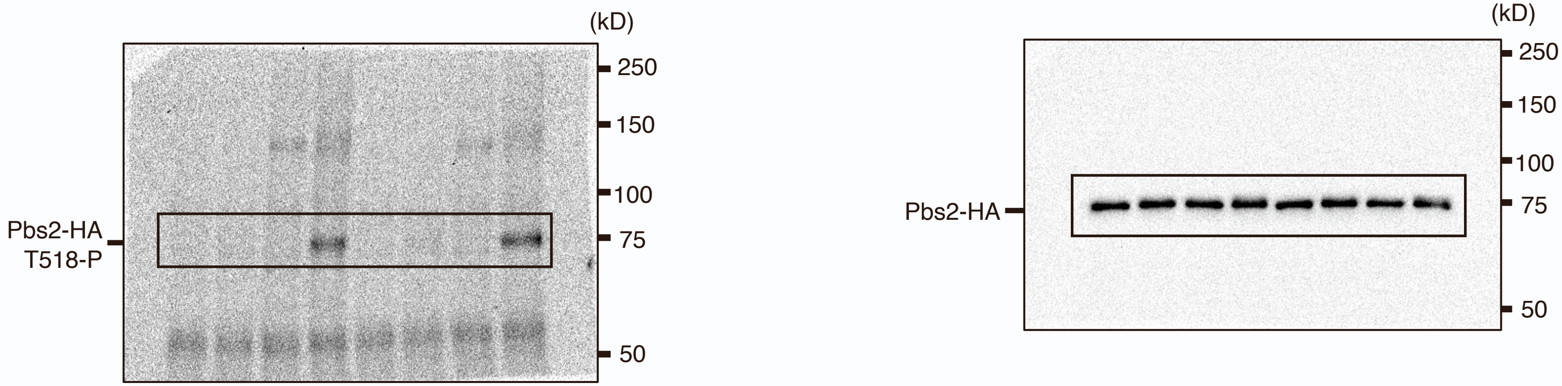

Figure 6B

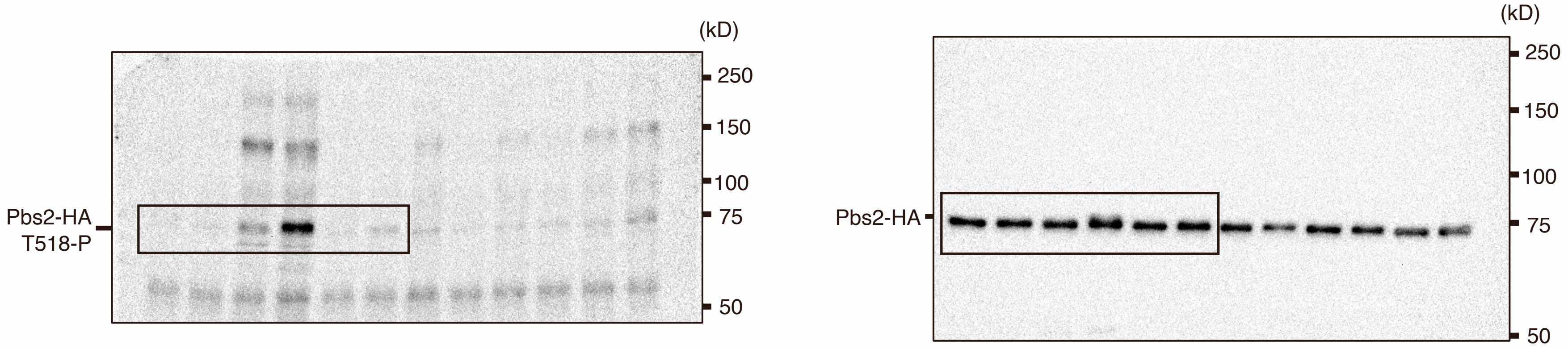

Figure 6C

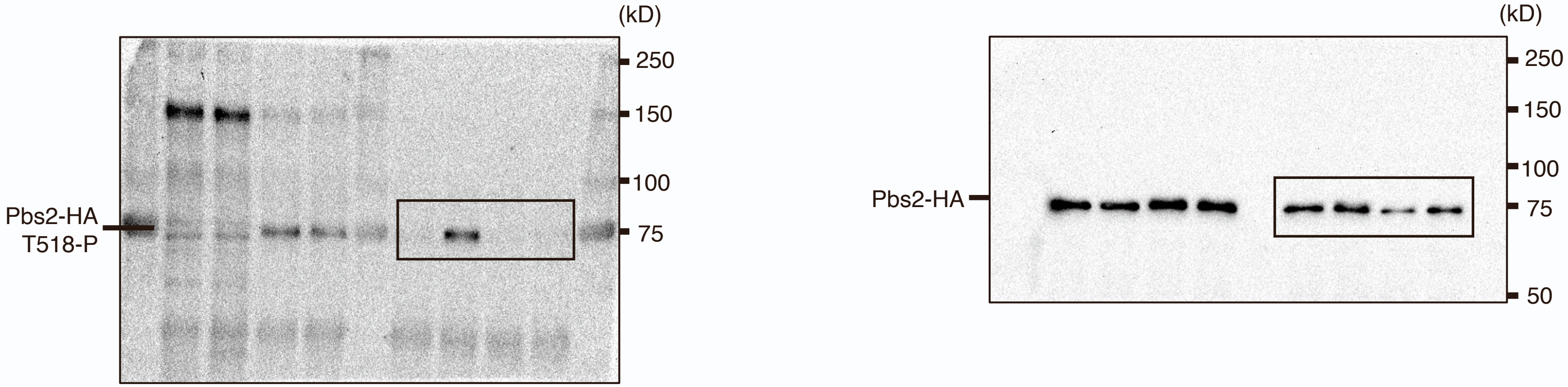

Figure 7A

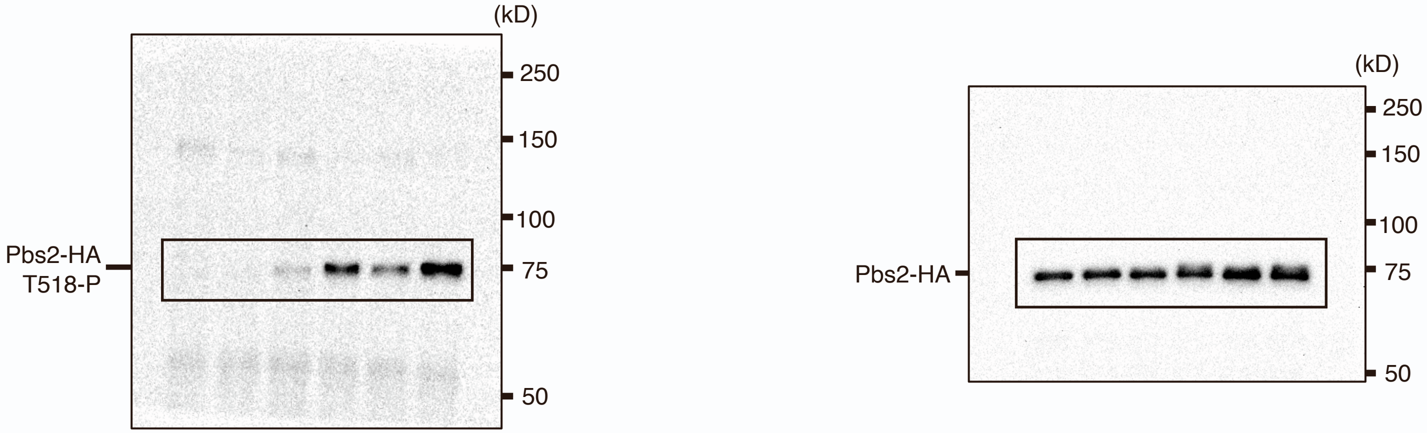

Figure 7B

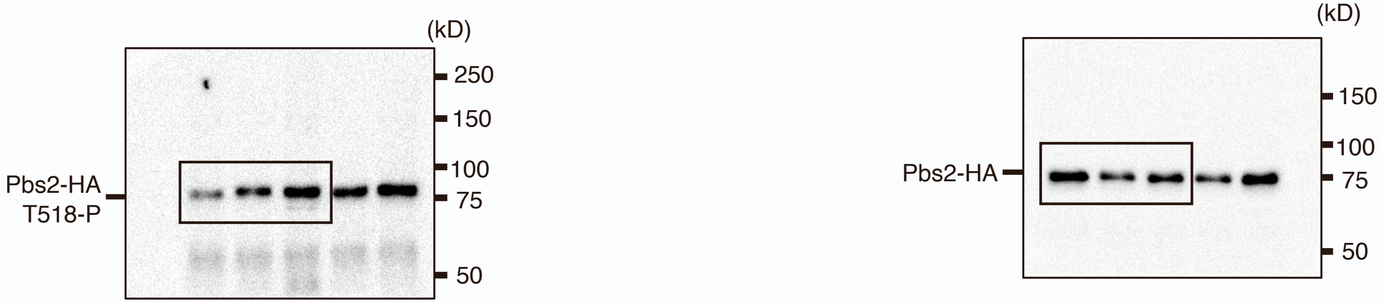

Supplement: Data S1. Uncropped western blot images [file mmc1.pdf]
